# How and Why the Cerebellum Recodes Input Signals: An Alternative to Machine Learning

**DOI:** 10.1177/1073858420986795

**Published:** 2021-02-09

**Authors:** Mike Gilbert, R. Chris Miall

**Affiliations:** 1 School of Psychology, University of Birmingham, Birmingham, UK

**Keywords:** cerebellum, coding, recoding, granular layer, mossy fiber, granule cell, Golgi cell, parallel fiber, Purkinje cell, theory

## Abstract

Mossy fiber input to the cerebellum is received by granule cells where it is thought to be recoded into internal signals received by Purkinje cells, which alone carry the output of the cerebellar cortex. In any neural network, variables are contained in groups of signals as well as signals themselves—which cells are active and how many, for example, and statistical variables coded in rates, such as the mean and range, and which rates are strongly represented, in a defined population. We argue that the primary function of recoding is to confine translation to an effect of some variables and not others—both where input is recoded into internal signals and the translation downstream of internal signals into an effect on Purkinje cells. The cull of variables is harsh. Internal signaling is group coded. This allows coding to exploit statistics for a reliable and precise effect despite needing to work with high-dimensional input which is a highly unpredictably variable. An important effect is to normalize eclectic input signals, so that the basic, repeating cerebellar circuit, preserved across taxa, does not need to specialize (within regional variations). With this model, there is no need to slavishly conserve or compute data coded in single signals. If we are correct, a learning algorithm—for years, a mainstay of cerebellar modeling—would be redundant.

## Introduction

Despite a rich and detailed literature, it remains unknown how the cerebellum processes input in order to select or generate output signals. The most popular model is that it implements a supervised learning algorithm (though it is not agreed which one). An important prediction of this idea is that Purkinje cells learn patterns stored as long-term modification of parallel fiber synapses. Synaptic weights are adjusted iteratively by training so that a subsequent repeat of input in a learned pattern^1^ is passed through a corresponding set of precision-graduated synaptic weights (Albus 1971; Brunel and others 2004; Dean and others 2010; Fujita 1982). The naive response of Purkinje cells to “raw” input is in this way displaced by learned output. The idea that pattern memory is stored as fine adjustments of parallel fiber synaptic transmission has for a long time been a strong influence on cerebellar theory (Raymond and Medina 2018).

We propose an alternative where learning provides timing but otherwise does not teach or control output, and recoding in the granular layer primarily has the function that it effectively strips out most of the variables contained in a barrage of mossy fiber input signals, effectively selecting some for a downstream effect and blocking an effect of others. It also converts input signals into internal group codes, that is, data are not contained in single signals but in firing of hundreds of co-active granule cells. Moreover, it is immaterial both which particular cells are active and which cells fire at what rates. In this proposal, synaptic learning does not displace control by rate codes, but instead polarizes synaptic transmission, so that individual adjustment of weights is absent.

To expand on this, it is intuitive to think of neural data as being coded in the activity of single cells—typically their firing rates or spike timing. However, where signals are processed in parallel these are not the only variables. For example, the number of active cells, the pattern (which are active and which are not), how they are distributed among target dendrites, how rates are distributed among active cells, and various aspects of timing, as well as group statistics derived from rates, such as the frequency distribution of rates in a defined population, and the range and mean, are all in theory capable of coding information.

We use “coding” to mean a variable attribute of a signal, or groups of signals, with a functional effect. There are many more variables than there are desirable effects, so that a variable with no function must be culled or confined to variation without an effect. It forms part of our argument that the granular layer is interposed between mossy fibers and Purkinje cells foremost to control unwanted variables. Control takes the same meaning as experimental control of variables—namely, they are eliminated or excluded from having an effect. This is not a modelling expedient but a physiological strategy.

**Figure 1.**
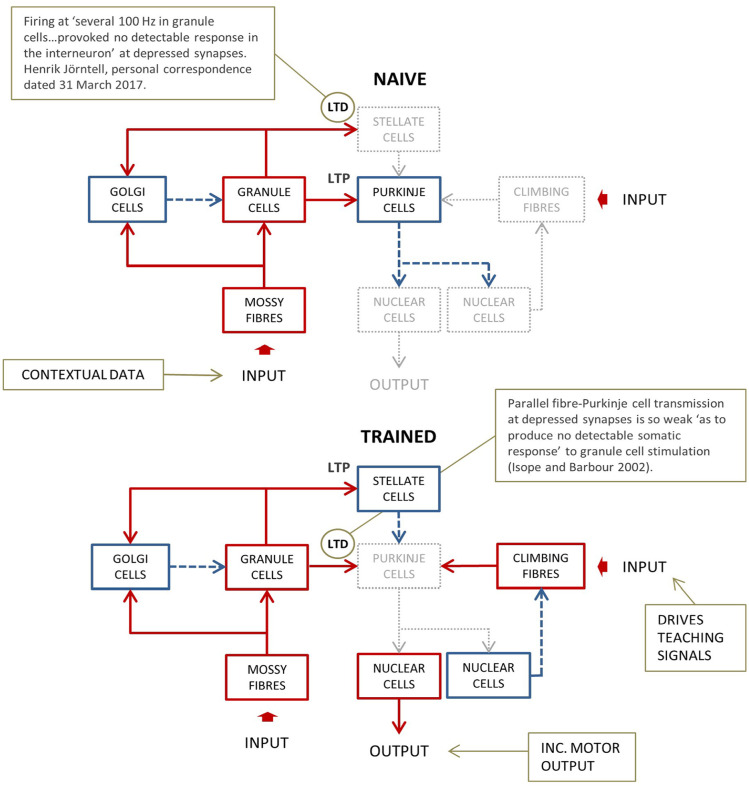
Schematic of cerebellar circuitry. The cerebellar cortex is divided into inner and outer layers, the granular layer and molecular layer respectively. Mossy fibers terminate in the granular layer, on granule cells, whose T-shaped axons rise into the molecular layer, where the cross-bars are all parallel and pass at right angles through the severely flattened Purkinje cell arbor, which fills the molecular layer vertically. Purkinje cells are organized functionally into long thin groups of several hundred cells termed microzones, also orthogonal to parallel fibers. Purkinje cells, though densely packed, are interleaved with inhibitory interneurons, also flattened, so that interneurons occupy the spaces between them (Palay and Chan-Palay 1974). Parallel fiber synaptic transmission is modified under climbing fiber tuition. (A) In the untrained state, granule cell transmission to Purkinje cells is robust and to stellate cells is very weak. Purkinje cell firing at high spontaneous rates, elevated by excitatory input, inhibits nuclear cells—the output cells of the circuit. Red boxes and solid arrows: active glutamate neurons; blue and dashed arrows: active GABA (γ-aminobutyric acid) neurons; gray and dotted arrows: silent neurons. (B) Training under instruction of climbing fibers (the conditions present in a conditioning protocol) reverses the sign of learning at both synapse types. Partly as a result, firing of Purkinje cells is weakened or suspended in the conditioned response, causing a phasic reduction of inhibition by Purkinje cells of nuclear cells.

Most input to the cerebellum terminates as mossy fibers that contact granule cells in the inner layer of the cerebellar cortex, the granular layer. (See Fig. 1 for a simplified circuit diagram.) The granule cell axon rises into the outer layer where it divides in two to give rise to parallel fibers (named because they lie parallel to the surface of the cerebellum and each other) which make contact in passing on Purkinje cells, on interneurons, which inhibit Purkinje cells, and on Golgi cells. The large Purkinje cell dendritic arbor is severely flattened in the plane orthogonal to parallel fibers, and intersected by some 350,000, of which around half make contact (Harvey and Napper 1991; Napper and Harvey 1988).

The anatomy of contact by mossy fibers on granule cells has long suggested that input to the cerebellum is recoded in the granular layer, generating coded patterns of parallel fiber signals activity. We argue that two internal codes can be simultaneously contained in the same group of granule cell signals and yet vary independently, so that they can be used in different functions at the same time without mutual interference. This permits Purkinje cells to learn cues coded in the binary pattern of active cells but for the response (i.e., the Purkinje cell firing rate) to be controlled separately. Counter to the traditional model, patterns are not remembered individually and graded synaptic weights are not used to derive output rates. As there is no need for graded weights, there is no need for a learning algorithm to teach them. Recoding converts the entire spectrum of uncontrolled variables contained in a mossy fiber bombardment into—at learned times—a binary code and a single group rate code, contained in recoded signals at the scale of input to a Purkinje cell.

Full circuit function involves more than just recoding within the granular layer. We do not claim to present a full model of circuit function. However, we recognize that some context is necessary to show how the ideas would fit into a feasible bigger picture. The section headed “Transmission of Rate Codes” and the “Discussion” section look at the implications for circuit function.

## Recoding: Patterns

Granule cells have three to five dendrites (average four) each of which receives contact from a single mossy fiber. Assuming contact is at random (i.e., mossy fibers do not individually select which granule cells they contact) it is very likely that each dendrite receives contact from a different mossy fiber (Supplementary Materials 1).

Mossy fiber terminals are ensheathed by a semipermeable membrane, in an arrangement termed a *glomerulus*, which also receives Golgi cell inhibition. Because neurotransmitter diffusion is restricted, as well as direct synaptic excitation of granule cells by mossy fibers, there is an additional, phasic component due to spillover, seen even with a single mossy fire action potential (Sargent and others 2005). Spillover of glutamate from neighboring granule cells increases the precision and reliability of the response and lengthens the window for temporal integration (Sargent and others 2005).

Each Golgi cell inhibits a large number of granule cells (Barmack and Yakhnitsa 2008; D’Angelo and others 2013). Inhibition is both synaptic and via GABA_A_ receptors located extrasynaptically (Brickley and others 1996; Nusser and others 1998). It is estimated that a large majority (98%) of inhibition of granule cells is through non-synaptic receptors (Duguid and others 2012).^2^ This extrasynaptic inhibition has a phasic component with a rise time of a few milliseconds (Mapelli and others 2014). Inhibition scales with local mossy fiber input rates (Duguid and others 2015).

The result is a competition between mossy fibers and Golgi cells for influence on granule cells, whose outcome depends partly on adjustments of the extrasynaptic neurotransmitter balance (Cesana and others 2013; Kanichay and Silver 2008), with a phasic component. We use “extrasynaptic” instead of “tonic” to avoid ambiguity, to include phasic and ambient components (we expand on fast-modulated phasic dynamics in Supplementary Materials 6). The outcome, we suggest, is a pronounced bias in the granule cell response, so that it either closely reflects mossy fiber input rates or there is no response. This is because the small minority that fire receive the strongest mean input rates, which are usually therefore individually the most competitive, and likely to dominate (Recoding: Firing Rates and Mossy Fiber–Granule Cell Transmission sections).

Mossy fibers branch terminally (in addition to collateralizing) and each branch ends in a cluster of terminals (average seven to eight terminals per cluster) (Shinoda and Sugihara 2013; Sultan and Heck 2003; Wu and others 1999). An estimated 100 mossy fibers terminate in a region the size of a cluster so that a cluster field contains an average of around 700 to 800 terminals (Sultan and Heck 2003). Each terminal contacts a single dendrite from each of what may be around 50 granule cells (Gao and others 2016; Jakab and Hámori 1988), although estimates vary (Ritzau-Jost and others 2014).

There is a minimum number of co-active mossy fibers needed to make a granule cell fire—thought to be three (Billings and others 2014; Jörntell and Ekerot 2006); we term this the *input threshold* and note that it is the count of active inputs, not their firing rates. Meeting the input threshold, while necessary, is not sufficient to make a granule cell fire. A mossy fiber signal must also individually be strong enough to compete robustly with Golgi cell inhibition.

As a number of Golgi cells inhibit a field, a granule cell may receive inhibition from a different Golgi cell to each of its dendrites. Inhibitory and excitatory rates received at a glomerulus are therefore a random pairing whose individual outcome—depolarization of the postsynaptic dendrite and charge transfer to the soma—is independent of other dendrites. We represent this as an adjustable probability of a glomerular inhibitory “veto.”

A winner-take-all outcome, or a functional equivalent, has empirical support. Activation of presynaptic GABA_B_ receptors on mossy fibers inhibits glutamate release (Mitchell and Silver 2000a), while activation of presynaptic mGluR2 receptors on Golgi cells inhibits GABA release (Mitchell and Silver 2000b). This would suggest that, at least over part of the range of input rates, positive feedback contributes to an amplified swing in the dominant direction. An outright winner, however, is unnecessary; the competition needs only to be independently contested at each glomerulus, with a field-wide outcome that is reliably predicted by input to that field. Whether or not there is always an emphatic result, this remains a tool to regulate numbers and not to adjust gain control.

If, as we propose, there is a fixed granule cell input threshold, the probability that a granule cell will meet threshold can be derived with a binomial from the ratio of active to inactive mossy fibers that supply a location. This gives the expected number of granule cells that fire because the law of large numbers holds that the ratio of the outcomes will converge toward the proportions predicted by their probability.

**Figure 2.**
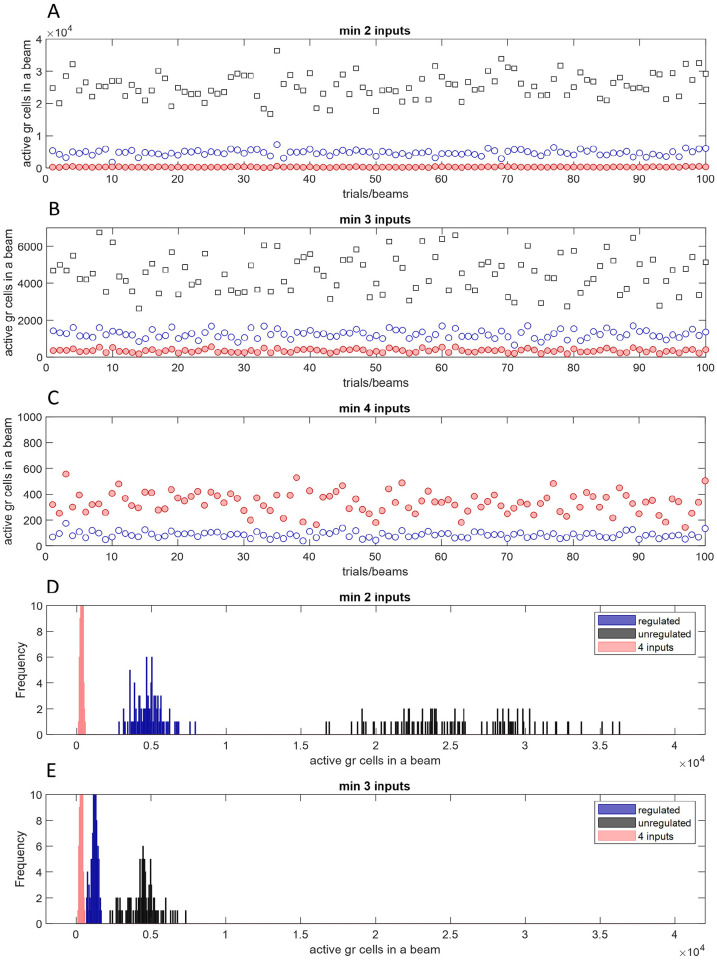
Estimate of the number of active parallel fibers that intersect a microzone. We simulated regulation of parallel fiber activity by Golgi cells to derive the total number of excited granule cells in a mediolaterally aligned row of 20 mossy fiber terminal cluster fields (together a “beam” measuring 3000 × 200 μm), trial by trial, in each of 100 trials. Each field receives input from a random number of mossy fibers in the range 3 to 30 and contains 8750 granule cells. The minimum number of mossy fibers needed to make a granule cell fire is 2 in panel A, 3 in B (the probable physiological input threshold), and 4 in C. Black squares are the estimated number of granule cells meeting the input threshold, disregarding Golgi cell regulation; blue circles show the number that fire subject to regulation by Golgi cells (a subset of the black data); and pink filled circles show the number which receive mossy fiber input to all 4 dendrites (regardless of Golgi cells). In panel C, the pink and black data are identical because (in that panel only) they represent the same thing; only pink are plotted. Note the pink data are identical in all graphs, for comparison. Note also y axes are scaled to the data, from a maximum of 40,000 in A down to 1000 in C. For comparison on the same scale, D and E are frequency distributions (bin size 50) of the same data as A and B. With an input threshold of 3 the average regulated number of active granule cells in a beam is approximately 1,200 (B: blue data). Of those, only a few tens receive 4 inputs (C: blue data). Note the maximum number even in A and D (~35,000) is still only 10% of the number of parallel fibers that pass overhead; 1200 represents around 0.003%. Estimated microzone dimensions vary—a length of 20 mm is uncontentious. One hundred trials shown in panels A to C can equally represent 100 beams—around the number that intersect a microzone.

Assuming Golgi cell inhibition of a glomerulus vetoes an effect of mossy fiber input with a probability P(v) , and x out of y mossy fibers are active, the probability that a particular dendrite receives excitatory input and no veto is (1−P(v))(x/y) . Given n = 4 dendrites per granule cell and an input threshold m , the expected number of granule cells in a cluster field (say f_1_) that fire out of a total N is

(1) $$E\left(f_{1}\right)=N\left(\begin{array}{l} \sum_{k=m}^{n}\frac{n!}{k!\left(n-k\right)!}*{\left(\left(1-P(v)\right)*\frac{x}{y}\right)}^{k}*\\ {\left[1-\left(\left(1-P(v)\right)*\frac{x}{y}\right)\right]}^{n-k} \end{array}\right)$$

where y is a constant (100, an estimate: Sultan and Heck 2003) and x varies for each trial, and for the purposes of simulation ranges between 3 and 30, generated randomly for each field. The total number of granule cells, N , is also a constant, and is taken as 8750, derived from convergent estimates (Supplementary Materials 2). We use this as part of a model to simulate regulation of parallel fiber activity by Golgi cells in Figure 2, to derive the homeostatically regulated number of active parallel fibers in a beam, and therefore the number in a remembered pattern of input to a Purkinje cell. A cross-section of a beam has the dimensions of the Purkinje cell arbor. (See Supplementary Materials 3 for the choice of dimensions of a beam.)

Golgi cell apical dendrites rise into and traverse the molecular layer, giving off few branches, where they receive contact from parallel fibers.^3^ As a result, a Golgi cell-mediated effect is exchanged by neighboring fields that are joined by parallel fibers, and in any row of fields there is an effect of each field on all others. The effect of any single mossy fiber signal is subject to a multiple chance variables in transit—which and how many granule cells receive it, which of those fire, how many (if any) Golgi cells receive the resulting granule cell signal, which field(s) they inhibit, and so on. Nonetheless, there is a predictable relationship of the density of parallel fiber activity with the probability of an inhibitory veto which we can quantify (we expand on this in Box 1), and a reciprocal relationship of P(v) with the density of activity (we discuss the timing of inhibition in Box 2).

BOX 1. **A Probability Loop: Mutually Regulating Probabilities**

The cerebellum, we propose, uses connected loops of mutually regulating probabilities to keep the volume of parallel fiber signals traffic to a fixed and narrow range. This permits contact between cells to be at random subject to a fixed probability for cells of that type. Inhibition of granule cells by Golgi cells is an example.

Contrary to the idea that a Golgi cells occupy separate compartments (Eccles and others 1967), their density (Llinás and Negrello 2015) and large, profusely ramified axonal field (Barmack and Yakhnitsa 2008) means that their territories overlap extensively. Contact on granule cells is at random, as far as is known, so that a granule cell may be inhibited with equal probability by any of the Golgi cells within that region, and indeed each of its dendrites may be inhibited by a different Golgi cell. Unitary recordings confirm that a granule cell receives inhibition from multiple Golgi cells (Duguid and others 2015).

How do Golgi cells, which each receive contact from a small random fraction of passing parallel fibers, control the number of granule cells that fire? We hypothesize that the effect of parallel fiber activity on Golgi cells is not the deterministic result of information coded in single parallel fiber signals but coded collectively in the density of parallel fiber activity. A Golgi cell need therefore receive contact from only a fraction of passing parallel fibers to respond proportionately and make in turn a proportionate adjustment to the probability of an inhibitory veto at mossy fiber–granule cell terminals. This then modulates the probability that a granule cell fires, creating a loop of mutually regulating probabilities.

Within this overarching control there is robust and important local modulation, driven by direct contact by mossy fibers onto the basal dendrites of Golgi cells, and by much more numerous contacts on Golgi cells from local than distant granule cells (Supplementary Materials 4).

It is unknown how many parallel fibers it takes to modulate firing of a Golgi cell. We assume that a low number is sufficient and the functional range is modest. Our choice is guided by the number and range for stellate cells. Excitation of a stellate cell is made up of “two to eight substantial EPSPs [excitatory postsynaptic potentials]” (Jörntell and Ekerot 2003, p. 9628). A Golgi cell resting potential near threshold is attested by the low number of mossy fibers (minimum four) sufficient for an effect (Kanichay and Silver 2008). We assume 4 to 16 parallel fiber inputs to the apical dendrites of a Golgi cell are active at any time. This reflects estimates that Golgi cells receive contact from around twice as many parallel fibers (1600: D’Angelo and others 2013) as do stellate cells. We use a sigmoidal function to translate the density of parallel fiber activity into the probability of a veto, with no effect of less than 4 parallel fiber inputs, and saturation of an effect above 16.

So, the cerebellum creates multiple loops of mutually regulating probabilities, and there is mutual regulation by the loops of each other. This is not a modeling expedient but a proposal—namely that circuit operation is designed to exploit the reliable outcome of probability at large numbers.

BOX 2. **Does Disynaptic Feedback Lag Behind Input Signals?**

The feedback loop mediated by Golgi cells (Box 1) is disynaptic, so that, on the face of it, we might expect feedback would lag the signals it was supposed to regulate. In fact, many granule cells (in Crus II) receive both direct and spillover-mediated sensory-evoked inhibition before excitation (Duguid and others 2015). Duguid and colleagues suggest that stimulation (an air puff to ipsilateral whiskers) may drive both fast input via the trigeminal nerve received by Golgi cells and slower input by way of the pons from the contralateral primary somatosensory cortex.

Fast superficial signals may provide the timing that mitigates feedback lag. There is vertical organization of mossy fiber input to the granular layer. In the C3 region in adult cats, cutaneously stimulated input is received by superficial granule cells, while input at deeper level is triggered by passive forelimb movement and joint flexion (Jörntell and Ekerot 2006, table 4) in the anesthetized animal. Input to the deepest level was undetected, suggesting it came via the pons from the cerebral cortex, which had been removed. Stratified topography is preserved in the molecular layer, with “granule cells in the inner granule cell layer giving rise to PFs [parallel fibers] in the inner molecular layer and granule cells in the outer granule cell layer giving rise to PFs in the outer molecular layer” (Zhang and Linden 2012, p. 122). There are exceptions, but this is the “prevalent rule” (Palay and Chan-Palay 1974, p. 66).

Most Golgi cells are concentrated in the superficial granular layer, immediately below the layer of Purkinje cell bodies that mark the boundary of the granular and molecular layers (Eccles and others 1967). Superficial Golgi cells differ from sparse, deeper cells in being bigger and having apical dendrites, which deeper cells lack. Fast superficial input would in theory permit feedback to provide timely regulation of signals received at deeper granular level after a longer transit time. This does not remove a delay at superficial level, but allows regulation at deeper level to compensate, to conserve a stable combined level of parallel fiber activity.

Excitatory input to both levels evoked by a common stimulus, or from different but functionally coupled sources, ensures local proportionality in a sagittal strip, as “sensory-evoked Golgi-cell inhibition scales proportionally with the level of excitatory mossy fiber synaptic input” (Duguid and others 2015, p. 13102). Proportionality is locally confined by anatomy. Mossy fibers terminate in a sagittal row of cluster fields (Sultan and Heck 2003), where they accordingly contact a sagittal strip of Golgi cells whose axonal fields extend, and overlap substantially, in the same direction (Barmack and Yakhnitsa 2008). Contact is both direct, on basal dendrites, and through local granule cells, which are much more likely to make contact than more distant granule cells (Supplementary Materials 4).^7^

A beam, crossing at right angles, lacks overall proportionality. In a beam, proportionality is in regional blocs (cluster fields in the model).

This suggests a broad division of integrated functions of inhibition, between locally driven inhibition, which is proportionate and timely, making the main contribution to maintain a fixed granule cell input threshold, and whole-beam regulation—by all fields of all other fields in a mediolateral beam—which lacks proportionality to local mossy fiber rates, but has the primary role in regulating parallel fiber activity.

Superficial Golgi cells conflate regulation i.e. both mechanisms act through the same Golgi cells. Control and action of deeper-lying Golgi cells is confined to the sagittal direction (because deeper-lying Golgi cells are under control exclusively of local mossy fiber innervation and contact from ascending axons of local granule cells).

The data in Figure 2 are derived by summing activity in a row of cluster fields and using it to calculate the influence of every field on each other, iterating the calculations until the activity stabilizes over a few iterations. Each iteration updates each field to reflect the activity of Golgi cells in neighboring fields. The Golgi cell axonal field is sagittally elongated, that is, in the direction of the long axis of a microzone—range 650 ± 179 µm by 180 ± 40 µm (in mice: Barmack and Yakhnitsa 2008)—and is the depth of the granular layer, which it fills vertically, so Golgi cells extend their plexus into (at least) the two sagittally neighboring cluster fields. We simulate five beams side by side to include an effect of neighboring beams on middle-beam activity.

**Figure 3.**
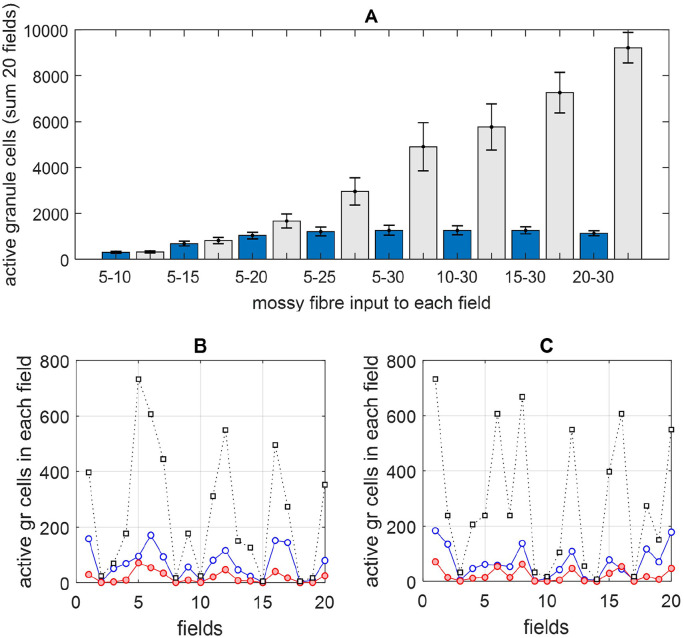
(A) The total number of active granule cells in a beam (a row of 20 fields) with and without Golgi cell regulation, averaged across 100 trials. Each field receives a random number of mossy fiber inputs in the range shown on the *x*-axis under the dark blue bars, which also applies for the light gray bar to the right. As the average number of inputs to a field increases, the number of granule cells that meet the input threshold steadily increases (light gray bars) but the regulated number—the total that fire—is held in a low range (dark blue bars). A minimum number of mossy fiber inputs to the beam as a whole is necessary for the regulated number to be capped. A minimum of a random number in the range 5 to 25 (out of 100) per field is sufficient. Between the ranges 5 to 25 and 15 to 30, the regulated number is stable. A higher range (20-30) causes the regulated number to fall slightly. This is because high input increases the probability that Golgi cells receive enough direct mossy fiber input for an effect. (B and C) The number of active granule cells in each field in a single trial. The color code is the same as Figure 2. A stable level of parallel fiber activity belies an uneven distribution of underlying granule cell activity. Even fields that receive the same number of inputs (so that the same number of cells meet the input threshold) do not necessarily contribute the same number of active cells to the parallel fiber total.

Each iteration also includes a field-by-field update for the higher probability that a Golgi cell receives contact from a granule cell in the same field than from a more distant granule cell (Supplementary Materials 4), and to reflect the fact that granule cell activity is unevenly distributed along a beam (Fig. 3). An adjustment is also made in each field to reflect the probability that a Golgi cell receives a direct effect of mossy fiber input to basal dendrites—a minimum of four inputs is necessary (Kanichay and Silver 2008).

The result—Figure 2—is that the number of active parallel fibers is confined to a low and narrow range (blue circles), varying much less than without Golgi cell feedback (black squares). As noted, it is thought that at least three out of three to five (typically four) mossy fibers that contact a granule cell must be active to drive firing. Certainly, one is too few (Bengtsson and Jörntell 2009; Chadderton and others 2004; Duguid and others 2012), while four would mean only a tiny fraction of granule cells ever meet threshold^4^ (red data, Fig. 2 all panels) even without the number then being further depressed by Golgi cells (Fig. 2C, blue circles).

The assumption of a fixed input threshold is a proposal, contrary to the long-standing idea (Marr 1969) that the function of Golgi cells is to *adjust* the threshold, providing the mechanism that keeps parallel fiber activity low. There is no direct evidence either that the threshold is fixed or that it is adjustable. Rather, we propose that a fixed threshold is necessary to maintain a predictable relationship of input to output of a field, and to prevent interference with high-fidelity mossy fiber-to-granule cell transmission, discussed in the section Transmission of Rate Codes.

Homeostatic regulation of parallel fiber activity so that it is maintained at a low level has a long history (Albus 1971; Billings and others 2014; Cayco-Gajic and others 2017; Cayco-Gajic and Silver 2019; Marr 1969). But the mechanism of regulation we propose is different. This is not a mere detail of implementation but necessary for the functions it has.

One of the functions is to eliminate an effect on granule cell firing rates of receiving a variable number of inputs, causing inconsistent translation of mossy fiber rates into granule cell rates. In the traditional model (Marr 1969), Golgi cells regulate activity by making adjustments to the number of mossy fibers needed to make a granule cell fire. In the present proposal, the function of Golgi cells is instead to ensure the needed number of inputs is fixed, not to adjust it. Without this, stronger mossy fiber rates would mean fewer inputs to a granule cell could make it fire.

A second—and the traditionally argued—function of recoding is to sparsen and decorrelate parallel fiber activity, to facilitate pattern storage. As that is well-covered in the literature we do not discuss it here. We only add that sparseness has the advantage that learning is confined to activated synapses (Wang and others 2000). By our estimate, the density of parallel fiber activity in a beam is maintained at about 1200 or so active parallel fibers (Fig. 2B and D). This is about 0.343% of 350,000 parallel fibers that are estimated to pass through a Purkinje cell dendritic field.

A third effect is control of pattern-related variables. The number and distribution of active parallel fibers are functionally fixed because density is constant and distribution is random. Decorrelation by recoding (Cayco-Gajic and others 2017) means that active parallel fibers are randomly distributed (and firing rates are randomly distributed among them). It follows that patterns do not in themselves differ in any way that affects the response of Purkinje cells. This has the functional outcome that Purkinje cells do not recognize patterns individually, their response discriminates only between the class of known patterns and the residual class of all other (therefore unknown) patterns. The only way that patterns differ is that they are highly input specific—each is effectively a unique response to a particular set of mossy fiber inputs to the system. From a cerebellar view, however, this is represented as generic notification only that mossy fiber activity is salient, defined by its repeated prior pairing with a climbing fiber signal. As there is not a variable effect of known patterns there is no interference by pattern memory with control of Purkinje cell rates. Conversely, granule cell firing rates do not affect storage or recognition of patterns. Rates are not reflected in synaptic weights (section Transmission of Rate Codes—although mossy fiber rates can be represented in the pattern of granule cells they excite—see section Recoding: Firing Rates), or remembered. To put that another way, group codes used in pattern matching and control of output firing rates vary independently.

## Recoding: Firing Rates

The previous section covered the constraint of parallel fiber activity to a stable proportion of parallel fibers. This section covers conversion of mossy fiber input rates into a collective statistical property of granule cell firing rates, at the numerical scale of input to a Purkinje cell.

**Figure 4.**
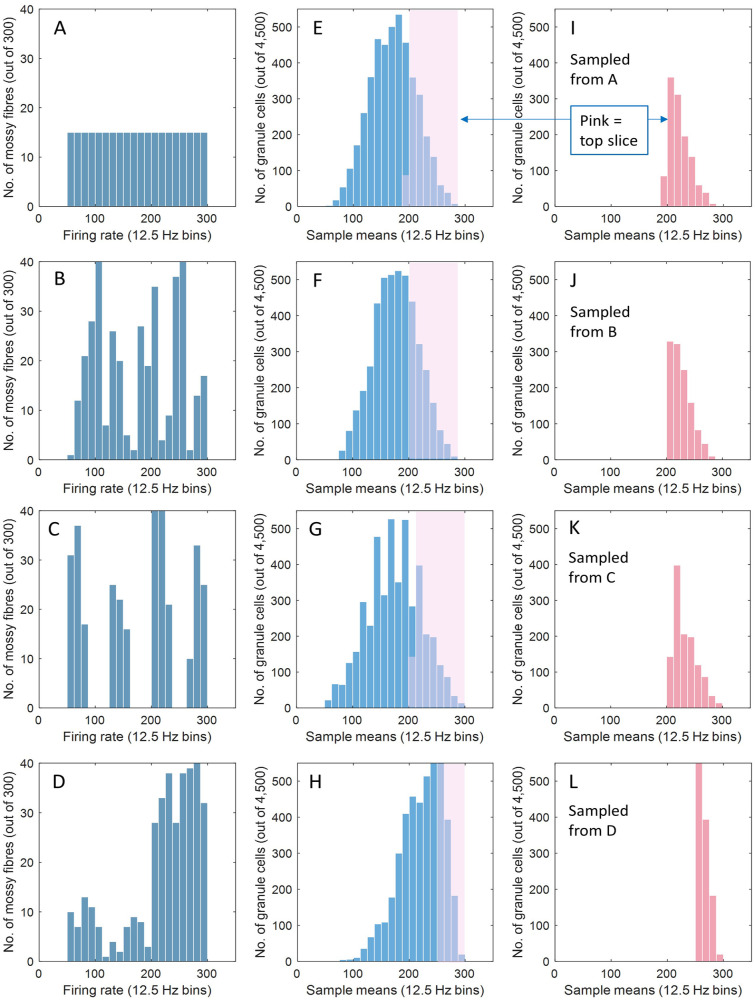
(A-D) Hypothetical examples of the frequency distribution of firing rates of 300 mossy fibers, in the range 50 to 300 Hz, typical of mossy fiber firing rates (Jörntell and Ekerot 2006 citing 29). In A the distribution is uniform, in B random, in C discontinuous, and in D weighted to the higher end of the range. The population of 300 is the average number of active mossy fibers out of the total of 2000 that innervate a mediolateral row of 20 mossy fiber terminal cluster fields (forming a 200 μm × 3 mm strip, or “beam”) in the Figure 2 simulation. (E-H) Frequency distributions of the mean mossy fiber rate received by each of 4500 granule cells, obtained by randomly sampling the A-D distributions, respectively. The sample size is three. The figure of 4500 is our previous estimate of the number of granule cells in a beam that receive contact from either three or four active mossy fibers (the large majority three, hence the sample size of three). The mossy fiber distributions in A-D are converted to an approximately normal distribution of the sample means. (I-L) Because of Golgi cell regulation, only a subset of these 4500 granule cells fire—the “top slice”: those that receive the highest mean rates of excitatory input. Each contains 1200 granule cells, the average number of active parallel fibers estimated in Figure 2B. The distribution of firing rates in this group is narrower than the mossy fiber range and the shape of the distribution is idiosyncratic and independent of the mossy fiber distribution.

Assuming contact by mossy fibers on granule cells is at random, granule cells randomly sample mossy fiber frequencies. Since, to fire, a granule cell must receive at least 3 inputs, and in our simulation the maximum is four the sample size is either three or four.^5^ The number of granule cells in a beam which receive that many is large (the black data in Fig. 2B), so that if the rates received by each granule cell are averaged, the frequency distribution of the sample means will approach a normal distribution by the central limit theorem (Fig. 4). This holds regardless of the number of mossy fiber inputs to the system, which ones are active, the rates they fire at, and how rates are distributed among active cells.

The number of samples in Figure 4 is 4500, the approximate number of granule cells that receive three or four inputs in Figure 2B (black data). Not all of these fire, however, because regulation by Golgi cells reduces the number. By our estimate, around 1200 fire out of 4500. The subset of granule cells that do fire are those that receive competitive mossy fiber rates to at least three dendrites. Inputs must be individually competitive because the outcome at each glomerulus is independent. As a result, the subset of granule cells that fire is predominantly made up of the high end of the frequency distribution of the sample means (Fig. 4I-L). The shape of the distribution in that range is highly constrained because it is always the upper range of a normal distribution (by the central limit theorem). We refer to mean rates in that range (and the granule cells that receive them, depending on the context) as the “top slice” of the distribution. The width of the top slice varies with the shape of the original distribution of mossy fiber rates received by the field as a whole. A wide range of (evenly distributed) mossy fiber rates results in a wider top slice. But even a wide range of mossy fiber rates is brought into a much sharper focus (Fig. 4D, H, and L). Also, the bottom-heavy shape of the top slice means most granule cells receive a mean input rate in a still narrower range, and the stable shape pinions the mean at a fixed point relative to the limits.

The top slice follows the mean of the whole distribution. If the whole distribution of the sample means moves to the right or left, the top slice follows, so that the mean of the top slice^6^ is constrained to a linear relationship with the mean of the distribution as a whole. Since the mean of the distribution is equal to the mean of the sampled population (i.e., of mossy fiber rates), again by the central limit theorem, it follows that the mean of the top slice has a linear relationship with the mean of mossy fiber rates received as input to the system (here, input to a beam).

Recoding in this way converts the entire gamut of uncontrolled variables contained in mossy fiber signals into a single group rate code received by Purkinje cells. (The translation of top slice statistics into granule cell rates is discussed in the next section.) The number of mossy fibers, the ones that are active, the permutation of rates they each fire at, and their distribution along a beam, are all unrepresented internally. Pattern size (the number of active cells, at the scale of input to a Purkinje cell) is fixed by homeostatic regulation. The make-up of the binary pattern of active granule cells, and which cells fire at what rates, though highly variable, are similarly both without an effect, as the statistically derived rate-coded attributes of parallel fiber activity are independent of the particular permutation of active cells, or how rates are distributed among them.

We note that the distribution of the sample means is not perfectly normal. A larger sample size would give a nearer-normal distribution. This raises the question: Why don’t granule cells each take a bigger sample? A possible reason is that a low number of inputs to granule cells is optimal for pattern separation and decorrelation (Cayco-Gajic and others 2017). Another possible reason may be that a larger number of short dendrites would increase the probability that the same mossy fiber would be resampled. However, a sample size of three to five is sufficient to transform the shape of the original distribution (the frequency distribution of mossy fiber rates) into a recognizably bell-like distribution of the sample means.

A potential effect of other “unwanted” variables is avoided by Purkinje cell dendritic anatomy. Contact of parallel fibers on Purkinje cells is exclusively on spines on thin tertiary Purkinje cell branches, so that a variable effect as a result of contact on spines or branches of different sizes does not occur (O’Brien and Unwin 2006; Wang and others 2000). Spine-bearing branchlets are distributed throughout the Purkinje cell dendritic field, which fills the molecular layer vertically, so activity throughout a beam is randomly sampled. Because input patterns are randomly decorrelated and large, an effect of chance variation of the distribution of dendritic contact on a Purkinje cell is absent. (See Supplementary Materials 5 for adaptations that prevent an effect of folding of the cerebellar cortex.)

Finally, it is worth restating that this should not be mistaken for a proposal that the function of recoding is to select strong signals per se. Rather, the function is that mossy fiber rates are faithfully and reliably converted into a group-coded attribute of granule cell rates (and other variables are fully controlled), or else transmission is blocked, and control of Purkinje cell rates is confined to that attribute (and other variables are fully controlled).

## Transmission of Rate Codes

In this section, we provide context so that recoding can be understood within the wider function of the circuit. We look in turn at mossy fiber–granule cell transmission, parallel fiber-Purkinje cell transmission (in the learned response), and inhibition of Purkinje cells by interneurons driven by parallel fibers.

### Mossy Fiber–Granule Cell Transmission

The mossy fiber–granule cell relay is highly adapted for precise and reliable transmission of high frequency signals (Delvendahl and Hallermann 2016; Rancz and others 2007; Ritzau-Jost and others 2014), despite receiving input to each dendrite only from a single afferent cell. For example, postsynaptic AMPA receptors at the mossy fiber–granule cell synapse operate in their linear range (Sargent and others 2005), show resistance to desensitization (DiGregorio and others 2007), and fast vesicle release and reloading facilitate transmission of rate coded information across a wide bandwidth of mossy fiber frequencies (Saviane and Silver 2006). Granule cells “have a relatively linear and uncomplicated conversion of depolarization level to spike rate” (Bengtsson and Jörntell 2009, p. 2393, citing Jörntell and Ekerot 2006 and D’Angelo and others 1998). We do not propose that granule cell rates are equal to mean input rates, only that transmission of the inputs that survive recoding obeys a relationship that is monotonic, reliable and proportional.

The model does not specify the exact mode of conversion of rate-coded input into granule cell rates. Our working hypothesis is that granule cells fire at a rate that is a function of the average of net excitation at each glomerulus (i.e., net of dilution by inhibition, if any), adjusted for short-term plasticity (Rancz and others 2007; Saviane and Silver 2006). Active granule cells are those that receive the strongest average, after competition. These are therefore a fair approximation of the top slice, because the top slice receives the highest excitatory rates. The approximation is improved by local coordination of Golgi cells by the mechanisms that regulate granule cell activity. Coordination may be enhanced through gap junctions that connect Golgi cell apical dendrites (Vervaeke and others 2012), because it allows excitatory charge to spread between cells. In theory, tightly aligned inhibition would more tightly limit active granule cells to the top slice, other things being equal, because it creates a “level playing field,” where the highest rates always win. High excitatory rates most strongly suppress glomerular inhibition, so we hypothesize that granule cell rates are good coders of high input rates, notwithstanding that there may be some inhibitory dilution.

That said, the shape of the frequency distribution of granule cell rates does not need to exactly mirror the top slice to conserve the proposed relationship with the wider distribution (or for good coding), as long as the shape is consistent.

Fidelity of transmission is facilitated by anatomy. Each granule cell dendrite receives contact from a single mossy fiber equidistant from the soma, so that there is no effect of the spatial distribution of inputs to a cell, because it does not vary. Also, a fixed input threshold means almost all granule cell signals are driven by the same number of mossy fiber signals, so that there is not a variable effect of the number of inputs, because that does not vary either. This does not preclude firing driven by a higher number of inputs but the numbers are very small (Fig. 2C, blue data).

### Excitation of Purkinje Cells in the Learned Response

Parallel fiber synaptic activation repeatedly paired with a convergent climbing fiber signal leads to long-term depression (Hansel and others 2001; Ito and Kano 1982; Qiu and Knopfel 2009). Depression is widespread and strong—in adult rats there is “no detectable somatic response” to granule cell stimulation at an estimated 80% to 85% of parallel fiber-Purkinje cell synapses (Isope and Barbour 2002, p. 9676). The Isope and Barbour detection threshold does not rule out a possible combined effect of multiple inputs. However, if so, input would be to synapses where plastic changes are randomly compounded because even modest numbers of stored randomly-decorrelated patterns overlap.

**Figure 5.**
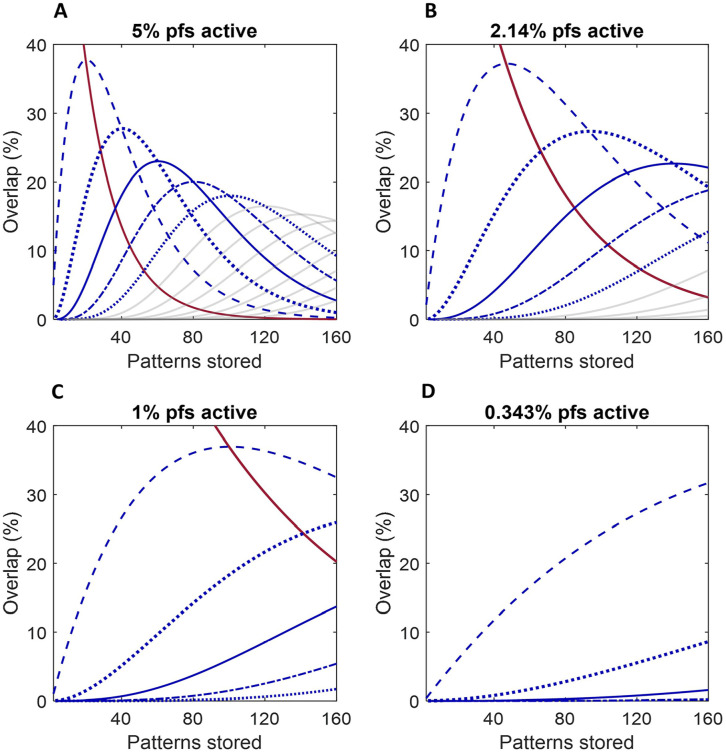
There is a statistically predictable frequency distribution of the fraction of parallel fiber synapses which participate in a remembered pattern that also participate in one other pattern, or in two, or in three or four, and so on. Overlap of a pattern with other patterns is always in the same proportions as all other patterns stored by the same Purkinje cell, and is also the same for all Purkinje cells trained to the same number of patterns, and therefore Purkinje cells in the same microzone. Moreover, where a synapse participates in more than one pattern (as most do), each synapse participates in an independent sample of other stored patterns. The changing relative proportions of a stored pattern, which overlap with other patterns, as more patterns are stored, is given by *y* = (*n*!/(*k*!(*n* − *k*))! * *p*^k^ * (1 − p)^(n − k)^, where *y* is the proportion of each pattern (the same for all of them) that overlaps with *k* other patterns, *n* + 1 is the total number of patterns stored, and *p* is the fraction of parallel fibers that are active. In reality, *p* is a constant, because the level of parallel fiber activity is confined by regulation to a narrow and stable range—that is, functionally fixed. For illustration, it is hypothetically varied from panels A to D to show the statistical pattern that emerges more clearly when more parallel fibers are active. The number of active parallel fibers out of 350,000 (the number estimated to pass through a Purkinje cell territory) is taken as 17,500 (5%), 7500 (2.14%), 3500 (1%), and 1,200 (0.343%) in A to D, respectively. Dashed line (all panels): the proportion of synapses that also participate in one other pattern. Thick dotted line: the proportion that also participate in two other patterns. Solid blue: three other patterns. Dots and dashes: four other patterns. Thin dotted: five other patterns. The pale gray lines in A and B show the proportion that participate in six other patterns, in seven, in eight, and so on, from left to right. The solid red line in A to C is the fraction of a set of modified synapses that does not participate in other patterns.

Overlap, and the seeming silence of most synapses, have attracted various model-based explanations which ring-fence the premise that synaptic weights are precision modified (Albus 1971; Brunel and others 2004; Dean and others 2010; Fujita 1982). Our explanation is that weights are not individually graded, but, rather, are all either feeble or fully functional, in each case with weights condensed into a narrow range, and that there is no redundancy. Patterns overlap in proportions predicted by a probability distribution (Fig. 5), so that they all each contain the same proportion of synapses, which also participate in no other patterns, or in one other, or two, and so on. As patterns are large enough to be received at a representative sample of weights, they are all received at a set of weights with the same frequency distribution and average. In other words, training does not teach a pattern-dependent response but rather ensures that the response does *not* discriminate between learned patterns. This suggests a generic effect of synaptic modification, rather than one that is pattern-dependent, at least in any way mediated by individually and selectively adjusted weights.

### Inhibition of Purkinje Cells in the Learned Response

The Purkinje cell dendritic arbor is severely flattened in the sagittal plane (orthogonal to parallel fibers) and interleaved with stellate cells, inhibitory interneurons that contact and inhibit Purkinje cells and receive excitatory input from parallel fibers. Paired stimulation of parallel fibers and climbing fibers potentiates the parallel fiber-stellate cell synapse (Jörntell and Ekerot 2003, 2011; Rancillac and Crépel 2004; Smith and Otis 2005). Therefore, while a known pattern is received exclusively at strongly depressed synapses on Purkinje cells it is received at viable synapses on stellate cells, driving inhibition of Purkinje cells.

Purkinje cell firing has a linear relationship with parallel fiber rates mediated through interneurons. In self-paced locomotion, firing of both molecular layer interneurons and Purkinje cells in the mouse was found to vary linearly and consistently with input rates. Interneurons reflect “granule cell input with linear changes in firing rate” (Jelitai and others 2016, p. 6), and “locomotion-dependent modulation of the balance between excitation and inhibition [of Purkinje cell dendrites] generates depolarizing or hyperpolarizing dendritic *V*_m_ [dendritic membrane voltage] changes that linearly transform into bidirectional modulation of PC SSp [Purkinje cell simple spike] output” (Jelitai and others 2016, p. 9). Granule cell firing rates can be highly variable. Jelitai and colleagues report the net effect of inputs (active during locomotion) on postsynaptic firing, and do not measure individual synaptic transmission. However, at the whole-cell level, the simple spike rate varies consistently and proportionately with afferent rates and this relationship is sufficient to explain the data.

In sum, at all nodes (mossy fiber–granule cell, granule cell–Purkinje cell, granule cell–stellate cell and stellate cell–Purkinje cell), there is an evidenced chain of transmission that is a function of rates, and in most cases a linear function.

## Discussion

We propose that recoding in the granular layer of the cerebellum generates two independently variable group codes that can be used in different functions at the same time without mutual interference. This permits *ad hoc* rate-coded control of Purkinje cell firing in a graded fashion gated by learned cues.

We argue that the primary function of recoding is to confine the response of Purkinje cells to an effect of some variables and not others. This has the functions both that it prevents interference with correct function by redundant variables, and of normalizing eclectic and modally diverse input signals, so that modular cerebellar circuits do not need to be adapted to the particular source or type of input they receive.

Contrary to the traditional model, learning does not have the function of precision-graduating synaptic transmission (so a learning algorithm with that function is unnecessary). The role of learning in control of Purkinje cells is almost the opposite, to eliminate *interference* of variable weights on rate coding, by causing synaptic transmission that is either very weak or robustly viable.

Learning also reverses the balance of input to a Purkinje cell in the learned response, from control by direct excitation to control by inhibition from interneurons, with learned timing provided by pattern memory. The theoretical attempt to explain a learning-modulated role of interneurons is nothing new (Albus 1971; Fujita 1982; Marr 1969), but here it is a switch that does not discriminate between learned patterns, and control is not by graded, intermediate adjustments of the balance. The effect of synaptic weights on transmission is state-dependent and not pattern-dependent. Learning, in this contention, does not specify or regulate output but gates it.

Monotonic transmission of rate codes is consistent with the finding that “the firing rate of many cerebellar neurons is a linear function of task related parameters . . . [and this] has been found at all levels of the cerebellar circuit” (Raymond and Medina 2018, p. 239). Strongly polarized synaptic weights reduce interference with transmission, so that data are not corrupted or lost at nodes of transmission. This is not to suggest parallel fiber weights are exactly binary, but rather that the net effect of learning on synaptic transmission is normalized at whole pattern/cell level. Even at the majority-depressed parallel fiber–Purkinje cell synapse, while the number of AMPA receptors is very severely depleted at some synapses, most are not completely devoid of receptors, and the number varies (Masugi-Tokita and others 2007)—consistent with weights that are in a depressed but not uniform state. However, recoding randomly distributes parallel fiber rates among active cells, so that any pattern-specific variation of weights is lost.

The redaction of redundant variables is necessary for function. The intractable-seeming problem of computing multidimensional input is one the brain has solved in the cerebellum by not doing it. The number of mossy fibers, the ones that are active, the permutation of rates they each fire at, and even the frequency distribution, are all functionless (to give examples). Internal signaling is group coded. Recoding even contrives that more than one code can be contained in the same group activity, and still vary independently—that is, each is insulated against an effect of the other.

The interposition of granule cells between mossy fibers and Purkinje cells is to remove an effect of input variables from the response to pattern recognition except for a chosen chain of effect. (This includes removing an effect of patterns themselves on output rates.) This is designed to cope with input diversity by ignoring it—meaning: There is no need for circuits to be differently adapted to the origin and type of signals they receive—removing the need to compute it. There are limited regional differences between circuits related to function, and input and output of a region is topographically (and often somatotopically) organized (Mottolese and others 2013; Shambes and others 1978), but within that circuits are not specialists. As a result, basic modular cerebellar circuit wiring has been enormously successful, and is broadly preserved across most taxa (Bell 2002). This is possible because recoding effectively normalizes input signals, rather than working with their differences. If we are correct, a learning algorithm—for years, a mainstay of cerebellar modeling—is unnecessary to explain the evidence.

## Supplemental Material

sj-pdf-1-nro-10.1177_1073858420986795 – Supplemental material for How and Why the Cerebellum Recodes Input Signals: An Alternative to Machine Learning Click here for additional data file. Supplemental material, sj-pdf-1-nro-10.1177_1073858420986795 for How and Why the Cerebellum Recodes Input Signals: An Alternative to Machine Learning by Mike Gilbert and R. Chris Miall in The Neuroscientist

## Notes

1 By “pattern” we mean the binary permutation of active cells. We assume this meaning unless a different meaning is clear from the context.

2 Where it is termed *tonic* without discriminating between phasic and ambient components.

3 This is not true for all Golgi cells but the bulk of them, whose cell bodies are found in the outer granular layer, close beneath the boundary between the granular and molecular layers formed by Purkinje cell bodies.

4 Except when a very high proportion of mossy fibers is active.

5 This is physiologically plausible, since although some granule cells have more than four dendrites the probability that a granule cell receives active input to five or more is extremely low.

6 So: the mean of the top-sliced sample means.

7 Local regulation by feedback, as opposed to direct input to Golgi cells from mossy fibers, is both via granule cell ascending axon contact (on both basal and apical dendrites of local Golgi cells) and parallel fiber contact of local granule cells (on apical dendrites of local Golgi cells).
