# Supplementary material for: How and Why the Cerebellum Recodes Input Signals: An Alternative to Machine Learning
Source: Neuroscientist. 2021 Feb 9;28(3):206–21. doi: 10.1177/1073858420986795 (PMC9136479; doi:10.1177/1073858420986795)
Supplement: sj-pdf-1-nro-10.1177_1073858420986795 – Supplemental material for How and Why the Cerebellum Recodes Input Signals: An Alternative to Machine Learning [file sj-pdf-1-nro-10.1177_1073858420986795.pdf]

SUPPLEMENTARY MATERIALS

1. Anatomical reasoning that contact on each of the dendrites of a granule cell is likely to be from a different mossy fibre

Mossy fibres terminate as clusters of terminals. An estimated 100 mossy fibres terminate in a region the size of cluster field. A mossy fibre gives rise to an estimated 7-8 terminals per cluster (Sultan and Heck 2003). Terminals intermingle. Granule cells have a small number of non-branching, short dendrites (15 µm, soma diameter 5-8 µm, so that a granule cell fits comfortably inside a cluster field). If we assume that each dendrite of a granule cell has an equal chance of contact with each of 7 x 100 mossy fibre terminals, the probability of an *n*th contact with the same mossy fibre is:

$$P_n = \left[\prod_{x=2}^{n}\left(\frac{m-x+1}{100m-x+1}\right)\right] \times \frac{y!}{x!\,(y-x)!}$$

where *m* is the number of terminals per cluster and *y* is the number of granule cell dendrites. The probability that a granule cell receives contact to each of its dendrites from a different mossy fibre is thus 1 minus the sum of the probabilities that it receives contact to any two or more dendrites from the same mossy fibre, or ~0.947. By this calculation around 95% of granule cells receive contact to each of their dendrites from a different mossy fibre, (and between 1 and 2 in a thousand receive contact from the same mossy fibre to 3 dendrites).

2. Convergent estimates of the number of granule cells in a cluster field

There are an estimated 1.92 x 10^6 granule cells per µl (in rats: Harvey and Napper 1988). The number of cluster fields which fit in that volume is $(1,000/150)^2$ x (1,000/200) = ~222, assuming cluster field dimensions of 150 x 150 x 200 µm (Sultan and Heck 2003). The number of granule cells in a cluster field is therefore 1,920,000/222 = 8,649. This is a good match with the estimate obtained in the text: if 100 mossy fibres each contribute 7 terminals to a cluster field (Sultan and Heck 2003) so that a cluster field contains 700 terminals, then the number of granule cells should be around 8,750 (700 terminals multiplied by 50 dendrites per terminal divided by 4 dendrites per granule cell).

3. The dimensions of a beam

The model calculates the number of active granule cells in a ‘beam’ of parallel fibres. The choice of the dimensions of a beam inevitably means defining a region which is in reality neither anatomically nor functionally discrete.

A beam represents a 3,000 x 200 µm folia-aligned slice of the cerebellar cortex, the horizontal dimensions of a row of 20 mossy fibre terminal cluster fields lined up in the mediolateral direction. The reason for basing it on a row of cluster fields is that estimates are available for the number of mossy fibres with input to a field and for terminals per cluster, and therefore terminals per field. The length of the simulated beam is half the span of parallel fibres, assuming a 6 mm parallel fibre span, so that all granule cells in the underlying granular layer give rise to parallel fibres that traverse the whole distance. A parallel fibre span of 6 mm is reported for cats, chickens and monkeys (Brand, Dahl et al. 1976, Mugnaini 1983); 5 mm is reported in rats (Harvey and Napper 1988, Harvey and Napper 1991).

The granule cells in a 3 mm beam in reality provide only half the parallel fibres that pass through it. Yet a longer beam would still need boundaries – no matter how long, there is always encroachment from granule cells which lie outside. Also, a larger population of parallel fibres in a beam would not mean that proportionately more are active, because the number is independent of the size of the population, for the same reason that it is independent of the number and distribution of inputs to the system, provided input is sufficient for regulation to reach equilibrium (Figure 3). (More input may be needed to reach equilibrium with a larger population.)

Granule cells have a distance-dependent strength of effect. The frequency of synapses declines along a parallel fibre with increasing distance from its bifurcation, such that there are half as many at its distal ends than at the centre (and synaptic densities are around half the size) (Pichitpornchai, Rawson et al. 1994). A variable effect of distance is not absent with a shorter beam, but the range of variation is smaller. We treat all granule cells as having the same strength of effect. By the same token the excluded effect of more distant granule cells is relatively weak.

4. Golgi cells receive most contact from local granule cells

Ascending axons of local granule cells make contact on both basal and apical dendrites of Golgi cells. A Golgi cell receives contact from around 1,600 parallel fibres, on apical dendrites (D'Angelo, Solinas et al. 2013). Around 400 are parallel fibres arising from local granule cells, which also make 400 ascending axon contacts (Cesana, Pietrajtis et al. 2013). Given the density of granule cells (Supplementary Materials 2) and parallel fibres (Harvey and Napper

1988), at a coarse estimate a Golgi cell receives contact from around 1 in 15 local cells, compared with around 1 in 300 for granule cells originating in other fields.

5. Adaptations to folding of the cerebellar cortex

References to 'density' of parallel fibre activity refer to the proportion of active cells. Density of parallel fibres themselves is affected by folding of the cerebellar cortex. It is higher in furrows and lower in peaks. In furrows the granular layer is thinner to compensate, and Purkinje cell territories are smaller, and in peaks the granular layer is thicker and Purkinje cells are larger. This has been estimated to ensure that Purkinje cells all receive contact from around the same number of parallel fibres (Eccles, Ito et al. 1967). A fixed proportion of active synapses is therefore also a fixed number.

6. Mossy fibre-granule cell transmission

In vitro, there is variation between trials, and, averaged, between synapses, of mean whole-cell recorded granule cell EPSCs evoked by mossy fibre stimulation (Sargent, Saviane et al. 2005). A similar result was later reported with mossy fibres which terminate on the same granule cell, leading to the proposal that modal pathway-specific synaptic properties are involved in coding granule cell signals (Chabrol, Arenz et al. 2015). It is not clear yet what we can infer from these results about normal function. In Sargent et al, stimulation is typically not above 2 Hz in order to avoid short-term depression observed at higher frequencies (Saviane and Silver 2006, Rancz, Ishikawa et al. 2007). Mossy fibres rate-code signals over a large bandwidth, typically in the hundreds of Hz (van Kan, Gibson et al. 1993, Rancz, Ishikawa et al. 2007). Synaptic properties observed with lower frequencies may not reflect glomerular dynamics in the behaving animal.

Another reason for caution is that the controlled conditions, which included a block of GABA, did not simulate extrasynaptic transmission with a physiological neurotransmitter balance. Extrasynaptic signalling may be an important form of excitatory glomerular transmission, as it is for inhibitory signals (Duguid, Branco et al. 2012, Mapelli, Solinas et al. 2014). In experimental conditions, variation is caused by trial-by-trial and mean differences in quantal size (*Q*), even at a single release site, and differences in the number of release sites (*N*) where there is a quantal event, and by release probability (Sargent, Saviane et al. 2005)[1]. As such, a variable effect may be absent from physiological function, where, at natural mossy fibre rates, rapid diffusion of spillover may homogenise the effect received by granule cells, mitigating uncertainty caused by *N* and *Q* and release probability. If the fast phasic component seen with GABA (Mapelli, Solinas et al. 2014) is mirrored with glutamate, the glomerular competition may importantly (and even primarily) reflect fast-modulated dynamics of extrasynaptic signalling, providing a smoothly but precisely time-varying signal to granule cells.

We see no reason that recoding cannot use modal signal properties to select which granule cells fire, or influence selection. The cerebellum receives modally diverse input. Within modal (Jörntell and Ekerot 2006) and topographical (Garwicz, Jörntell et al. 1998, Jörntell and Ekerot 2003) organisation, information of different types is mixed, even onto the same granule cell (Huang, Sugino et al. 2013, Chabrol, Arenz et al. 2015). Still, there is order. Input onto a granule cell is not randomly sourced. For example, in the C3 region, mossy fibre input to the superficial granular layer of a microzone is modally restricted to cutaneous sensory signals

[1] *Q* is 'the postsynaptic response to a single vesicle', and *N* = 200-400 at a single mossy fibre terminal, shared among perhaps 50 granule cells (as noted in the main text, estimates vary).

and topologically limited to a discrete and often small region of the body surface (Garwicz, Jörntell et al. 1998, Apps and Garwicz 2005). Input to granule cells is therefore in somatosensory sub-modalities (Jörntell and Ekerot 2006) activated by conditions at the same site. If this organisation is typical generally, it would be a viable strategy to exploit modal variability of pathways (or of signal characteristics) to use granule cells to detect which submodality is triggered, or which combination of submodalities.

REFERENCES

Apps, R. and M. Garwicz (2005). "Anatomical and physiological foundations of cerebellar information processing." Nat Rev Neurosci **6**(4): 297-311.
Brand, S., A. L. Dahl and E. Mugnaini (1976). "The length of parallel fibers in the cat cerebellar cortex. An experimental light and electron microscopic study." Exp Brain Res **26**(1): 39-58.
Cesana, E., K. Pietrajtis, C. Bidoret, P. Isope, E. D'Angelo, S. Dieudonne and L. Forti (2013). "Granule cell ascending axon excitatory synapses onto Golgi cells implement a potent feedback circuit in the cerebellar granular layer." J Neurosci **33**(30): 12430-12446.
Chabrol, F. P., A. Arenz, M. T. Wiechert, T. W. Margrie and D. A. DiGregorio (2015). "Synaptic diversity enables temporal coding of coincident multisensory inputs in single neurons." Nat Neurosci **18**(5): 718-727.
D'Angelo, E., S. Solinas, J. Mapelli, D. Gandolfi, L. Mapelli and F. Prestori (2013). "The cerebellar Golgi cell and spatiotemporal organization of granular layer activity." Front Neural Circuits **7**: 93.
Duguid, I., T. Branco, M. London, P. Chadderton and M. Hausser (2012). "Tonic inhibition enhances fidelity of sensory information transmission in the cerebellar cortex." J Neurosci **32**(32): 11132-11143.
Eccles, J. C., M. Ito and J. Szentágothai (1967). The cerebellum as a neuronal machine. Berlin, New York etc., Springer-Verlag.
Garwicz, M., H. Jörntell and C. F. Ekerot (1998). "Cutaneous receptive fields and topography of mossy fibres and climbing fibres projecting to cat cerebellar C3 zone." J Physiol **512 (Pt 1)**: 277-293.
Harvey, R. J. and R. M. Napper (1988). "Quantitative study of granule and Purkinje cells in the cerebellar cortex of the rat." J Comp Neurol **274**(2): 151-157.
Harvey, R. J. and R. M. Napper (1991). "Quantitative studies on the mammalian cerebellum." Prog Neurobiol **36**(6): 437-463.
Huang, C. C., K. Sugino, Y. Shima, C. Guo, S. Bai, B. D. Mensh, S. B. Nelson and A. W. Hantman (2013). "Convergence of pontine and proprioceptive streams onto multimodal cerebellar granule cells." Elife **2**: e00400.
Jörntell, H. and C. F. Ekerot (2003). "Receptive field plasticity profoundly alters the cutaneous parallel fiber synaptic input to cerebellar interneurons in vivo." J Neurosci **23**(29): 9620-9631.
Jörntell, H. and C. F. Ekerot (2006). "Properties of somatosensory synaptic integration in cerebellar granule cells in vivo." J Neurosci **26**(45): 11786-11797.
Mapelli, L., S. Solinas and E. D'Angelo (2014). "Integration and regulation of glomerular inhibition in the cerebellar granular layer circuit." Front Cell Neurosci **8**: 55.
Mugnaini, E. (1983). "The length of cerebellar parallel fibers in chicken and rhesus monkey." J Comp Neurol **220**(1): 7-15.

Pichitpornchai, C., J. A. Rawson and S. Rees (1994). "Morphology of parallel fibres in the cerebellar cortex of the rat: an experimental light and electron microscopic study with biocytin." J Comp Neurol **342**(2): 206-220.
Rancz, E. A., T. Ishikawa, I. Duguid, P. Chadderton, S. Mahon and M. Hausser (2007). "High-fidelity transmission of sensory information by single cerebellar mossy fibre boutons." Nature **450**(7173): 1245-1248.
Sargent, P. B., C. Saviane, T. A. Nielsen, D. A. DiGregorio and R. A. Silver (2005). "Rapid vesicular release, quantal variability, and spillover contribute to the precision and reliability of transmission at a glomerular synapse." J Neurosci **25**(36): 8173-8187.
Saviane, C. and R. A. Silver (2006). "Fast vesicle reloading and a large pool sustain high bandwidth transmission at a central synapse." Nature **439**(7079): 983-987.
Sultan, F. and D. Heck (2003). "Detection of sequences in the cerebellar cortex: numerical estimate of the possible number of tidal-wave inducing sequences represented." J Physiol Paris **97**(4-6): 591-600.
van Kan, P. L., A. R. Gibson and J. C. Houk (1993). "Movement-related inputs to intermediate cerebellum of the monkey." J Neurophysiol **69**(1): 74-94.
